# Intraocular Pressure-Lowering Effects of Trabeculectomy Versus MicroShunt Insertion in Rabbit Eyes

**DOI:** 10.1167/tvst.10.9.9

**Published:** 2021-08-06

**Authors:** Tomokazu Fujimoto, Kei-Ichi Nakashima, Fumika Watanabe-Kitamura, Takahiro Watanabe, Kenichi Nakamura, Kentaro Maki, Atsushi Shimazaki, Masatomo Kato, Hidenobu Tanihara, Toshihiro Inoue

**Affiliations:** 1 Department of Ophthalmology, Faculty of Life Sciences, Kumamoto University, Kumamoto, Japan; 2 Santen Pharmaceutical Co., Ltd., Osaka, Japan; 3 Director of Kumamoto University Hospital, Kumamoto University, Kumamoto, Japan

**Keywords:** intraocular pressure, PRESERFLO microshunt, trabeculectomy, RNA-seq

## Abstract

**Purpose:**

To compare the surgical results of PRESERFLO MicroShunt (MicroShunt) insertion and trabeculectomy in rabbit eyes.

**Methods:**

Trabeculectomy or MicroShunt insertion was performed on the eyes of Japanese white rabbits. Intraocular pressure (IOP) was measured on conscious rabbits using a rebound tonometer for up to 12 weeks after surgery. Filtering bleb appearance was evaluated. Scarring in the filtering bleb was assessed by immunohistochemical analyses. The change in mRNA expression in the conjunctiva was evaluated using RNA sequence analyses.

**Results:**

The preoperative IOP of the operative eye did not differ significantly between trabeculectomy (11.6 ± 1.0 mmHg, *n* = 10) and MicroShunt insertion (12.6 ± 1.3 mmHg, *n* = 10). In both groups, the IOP of the operative eye was significantly lower than that of the contralateral eye at one day postoperatively, which continued until 12 weeks after surgery. The peak differences in IOP were −8.4 ± 3.0 (trabeculectomy) and −8.1 ± 2.1 mmHg (MicroShunt) at two weeks after surgery; no significant differences were observed in IOP reduction between the groups. Appearance and immunohistochemical analyses of the filtering bleb showed no significant difference between the groups. Moreover, RNA sequence analysis results showed no difference between the groups in mRNA expression fluctuations.

**Conclusions:**

Postoperative IOP, bleb appearance, and immunohistochemical analysis results were similar in the trabeculectomy and MicroShunt groups, indicating that MicroShunt insertion is as effective as trabeculectomy in lowering IOP.

**Translational Relevance:**

Comparison of surgical procedures using animal models has made it possible to predict clinical efficacy and safety.

## Introduction

Glaucoma is a leading cause of blindness,^1^ and its progression is associated with elevated intraocular pressure (IOP),^2^^,^^3^ cardiovascular dysfunction,^4^^,^^5^ genetic factors,^6^ oxidative stress,^7^^,^^8^ blood-flow reduction in the optic nerve,^9^ and enlargement of the gap between cerebrospinal fluid pressure and IOP.^10^ Elevated IOP has long been associated with glaucoma; as such, the only effective treatment for inhibiting visual field defect progression is IOP-lowering therapies.^11^^,^^12^ Trabeculectomy is a well-known glaucoma surgery used to reliably reduce IOP.^13^ However, this treatment requires a high level of skill and surgical experience to achieve an adequate postoperative IOP. Various glaucoma drainage devices have recently been developed; their reported IOP-reduction effects are comparable to those associated with trabeculectomy.^13^^–^^17^ The PRESERFLO MicroShunt (“MicroShunt,” Santen Pharmaceutical, Osaka, Japan) consists of an 8.5-mm-long tube, with a lumen diameter of 0.07 mm made entirely of poly(styrene-block-isobutylene-block-styrene) (SIBS).^18^ The first use of SIBS in the body was as a coating on the TAXUS drug-eluting coronary stent (Boston Scientific Corp., Natick, MA).^19^ TAXUS has been found to be highly inert and safe in the body, with implant use dating back to the year 2000.^20^ In addition, according to a rabbit model study, SIBS is less likely than silicone rubber controls to cause conjunctival scarring.^21^

The MicroShunt is a glaucoma drainage device that drains aqueous humor to a subconjunctival/subTenon's capsule flap.^22^^,^^23^ Although the method of MicroShunt insertion shares some similarities to trabeculectomy (i.e., subconjunctival/Tenon's dissection and use of mitomycin C), it does not require the creation of a scleral flap or sclerectomy/peripheral iridectomy procedures.^23^ Trabeculectomy regulates aqueous outflow by controlling suture tension of the scleral flap, whereas the MicroShunt regulates aqueous outflow by flow resistance, which is a function of the lumen and length of the tube. Thus, MicroShunt implantation can provide similar results to trabeculectomy without relying on precise surgical suture-tensioning techniques practiced by experienced clinicians. Although short-term postoperative results of MicroShunt insertion have been reported,^23^^–^^25^ no studies have compared the results of bleb morphology, immunohistochemical analyses, or gene expression after MicroShunt insertion versus trabeculectomy. It is not possible in actual clinical practice to directly remove and evaluate filtration blebs. Therefore, we compared the surgical results of MicroShunt insertion and trabeculectomy in the rabbit eye, with a focus on IOP, bleb morphology, immunohistochemical analyses, and gene expression outcomes.

## Materials and Methods

### Animal Experiment

Twelve- to 14-week-old Japanese white rabbits (KBT Oriental, Saga, Japan) were used for the animal experiments. All experiments were conducted according to the Association for Research in Vision and Ophthalmology Statement for the Use of Animals in Ophthalmic and Vision Research and were approved by the Animal Care and Use Committee at Kumamoto University. Trabeculectomy and MicroShunt insertion were performed in 10 rabbits each, and the same animals were subjected to IOP measurement and bleb observation for up to 12 weeks after surgery. IOP was measured at one day before surgery and at one day and 1, 2, 4, 6, 8, 10, and 12 weeks postoperatively using TONOVET (Icare, Helsinki, Finland) in conscious rabbits. Aqueous humor and eyeballs were collected at 12 weeks after the operation for immunostaining and cytokine concentration measurements. An additional 24 animals were operated on for immunostaining and cytokine concentration measurements at two, four, and eight weeks after surgery, and four samples from each group were collected at each time point. In addition, nine animals underwent MicroShunt insertion for RNA-seq analysis at 3 hours, 3 days, and 14 days after surgery, and three samples of conjunctival tissue including filtering bleb, were collected from each group. The times required for individual trabeculectomy and MicroShunt insertion procedures were recorded.

### Trabeculectomy

Trabeculectomy was conducted as described previously,^26^ with minor modifications. Rabbits were anesthetized by intramuscular injection of ketamine hydrochloride (50 mg/kg; Daiichi Sankyo Propharma, Tokyo, Japan) and xylazine hydrochloride (10 mg/kg; Bayer HealthCare, Leverkusen, Germany). The right eye was washed with polyvinyl alcohol iodine (Nitten Pharmaceutical, Nagoya, Japan), and an eyelid speculum was used to expose the operating space. A 7 mm conjunctival incision was made along the corneal limbus. A triangular scleral flap with a side length of 3 mm was made along the corneal limbus in the upper sclera. Three sponges soaked with mitomycin C (MMC; 0.4 mg/mL; Kyowa Kirin, Tokyo, Japan) were applied under conjunctival and scleral flaps for three minutes. Then the subconjunctival tissue and scleral flap were washed with 10 mL normal saline (Otsuka, Tokyo, Japan). The trabecular meshwork below the scleral flap was removed in a rectangular shape with a Feather scalpel. The iris was pulled from the excisional wound, and peripheral iridectomy was performed. The scleral flap was sewn to the sclera using 10-0 nylon at a tension that did not completely close the flap. Then the conjunctival wound was closed with 10-0 nylon. Finally, a conjunctival injection of 0.1 mL betamethasone (0.4% RINDERON, Shionogi, Osaka, Japan) was performed, and ointment containing erythromycin and colistin (Ecolicin Ophthalmic Ointment, Santen Pharmaceutical Co., Ltd., Osaka, Japan) was applied. No additional medications were used postoperatively.

### MicroShunt Insertion

PRESERFLO MicroShunt devices were provided by Santen Pharmaceutical. Except for the creation of a scleral flap, the MicroShunt implant procedure was performed in the same manner as the trabeculectomy procedure, up to the point of MMC application. Once the sclera was exposed, a point 3 mm from the limbus was marked, and a shallow 1 mm × 1 mm scleral pocket was incised with a slit knife (Mani, Tochigi, Japan) directed towards the limbus. Then a 25-G needle tract was made connecting the scleral pocket to the anterior chamber. The MicroShunt was inserted through the scleral pocket and needle tract, with the fins of the MicroShunt wedged firmly into the scleral pocket. The entrance to the MicroShunt bisected the angle between the cornea and the iris. After MicroShunt fixation, the conjunctival wound was closed with a 10-0 nylon suture. Following closure, a conjunctival injection of 0.1 mL betamethasone (0.4%) was performed, and ointment containing erythromycin and colistin was applied. No additional medications were used postoperatively.

### Bleb Morphological Evaluation

Photographs of each bleb were taken with a digital camera (COOLPIX S9900, Nikon, Tokyo, Japan), and bleb area and vascularity were classified using our own evaluation criteria (Supplementary Figures S1 and S2), which are based on Moorfields Bleb Grading System.^27^ Bleb area and vascularity were evaluated by three ophthalmologists using blinded bleb-photograph data.

### Immunohistochemical Analyses

Immunostaining was performed using a previous method with some modifications.^26^^,^^28^ The enucleated eyes were cut in half, and the lens and vitreous were removed. Then the eyes were fixed in Super Fix (Kurabo, Osaka, Japan) overnight at 4°C and then soaked in 30% sucrose (FUJIFILM Wako Pure Chemical, Osaka, Japan) for 48 hours at 4°C. Then they were trimmed and embedded in an optimum cutting temperature compound (Sakura Finetek, Torrance, CA) and cut into vertical frozen sections (thickness: 8 µm). After blocking endogenous peroxidase activity with 3% hydrogen peroxide in methanol, the sections were blocked with 10% goat serum for 30 minutes at room temperature, and then incubated with goat anti-α-smooth muscle actin (α-SMA) antibody (1:1000; Dako, Kyoto, Japan) or mouse anticollagen type 1 antibody (1:200; Abcam, Cambridge, UK) for 30 minutes at room temperature. Following washing with phosphate buffered saline, the sections were incubated with secondary antibody (Simple Stain Rat MAX-PO; Nichirei Biosciences, Inc., Tokyo, Japan) for 30 minutes at room temperature. As a chromogenic substrate, 3,3'-diaminobenzidine tetrahydrochloride (DAB Peroxidase Substrate Kit; Vector, Burlingame, CA) was used. Hematoxylin was used as a counterstain. The samples were observed under an all-in-one epifluorescence microscope (BZ-X710; Keyence, Osaka, Japan); photographs of whole sections were created by combining multiple photos using the accompanying software (BZ-H3A, Keyence). We performed immunostaining on at least three eyes at all time points.

### Measuring the MCP-1 and IL-6 Concentrations

The concentrations of monocyte chemotactic protein-1 (MCP-1) and interleukin-6 (IL-6) in aqueous humor were measured using ELISA kits (MCP-1; SEA087Rb, IL-6; SEA079Rb; Cloud-Clone Corp., Katy, TX), according to the manufacturer's protocol. The absorbance at 450 nm was measured using a microplate reader (Multiskan FC; Thermo Fisher Scientific, Rockford, IL).

### RNA Sequence Data of Bleb Conjunctiva

For the sequence data of trabeculectomy samples, the previously analyzed data (GEO accession number, GSE156781) were used, and comparative analyses with MicroShunt were performed. RNA was isolated from rabbit conjunctiva, including filtering blebs, using TRIzol reagent, Phasemaker Tubes, and a PureLink RNA Mini Kit (Thermo Fisher Scientific), according to the manufacturer's instructions. Quality control of RNA samples was conducted using a NanoDrop system (Thermo Fisher Scientific) for quantification and an Agilent 2200 TapeStation (Agilent Technologies, Santa Clara, CA) for quality assessment; samples with an RNA integrity number of 7 or higher were used for further analyses. For library preparation, TruSeq Stranded mRNA Library Prep (Illumina, San Diego, CA), IDT for Illumina-TruSeq RNA UD indexes (Illumina), and an Agilent XT Auto System (Agilent Technologies) were used, according to the manufacturer's instructions (TruSeq Standard mRNA Reference Guide v00). Final libraries were quality checked on an Agilent 2100 Bioanalyzer (Agilent Technologies) and were run on a NovaSeq 6000 (Illumina), according to the manufacturer's protocol. The 150 bp paired-end reads were mapped to the rabbit genome sequence using OryCun2.0 (https://www.ncbi.nlm.nih.gov/assembly/GCF_000003625.3/) and STAR. Expression levels were calculated as the counts per million mapped reads (CPM) of each gene and each transcript, based on the mapping information and the gene locus, using the GeneData Profiler Genome (GeneData, Basel, Switzerland). R (ver. 3.4.1) and edgeR (ver. 3.20.9) programs (R Foundation for Statistical Computing, Vienna, Austria) were applied to determine differential gene expression analyses. An adjusted *P*-value cutoff (q ≤ 0.05) was used to determine the significance of differential gene expression. We also performed gene ontology (GO) enrichment analyses for the differentially expressed genes obtained from the surgical samples using Database for Annotation, Visualization, and Integrated Discovery (DAVID 6.8; https://david.ncifcrf.gov/).

### Statistical Analyses

Statistical analyses were performed using JMP statistical software (version 14.3.0; SAS Institute, Cary, NC), except for the RNA sequence data. The IOP, MCP-1, and IL-6 data were analyzed using Student's *t*-test. The bleb area and vascularity were analyzed using the Wilcoxon rank sum test, and differences were considered statistically significant at *P* < 0.05. All data are shown as mean ± standard deviation.

## Results

### Comparison of Operation Times between Trabeculectomy and MicroShunt Insertion

The average operation times were 30.6 ± 2.26 minutes for trabeculectomy (*n* = 8) and 14.8 ± 1.04 minutes for the MicroShunt (*n* = 8). The operation time of MicroShunt insertion was significantly shorter than that of trabeculectomy (*P* < 0.0001).

### IOP Reduction after Operation

**Figure 1.**
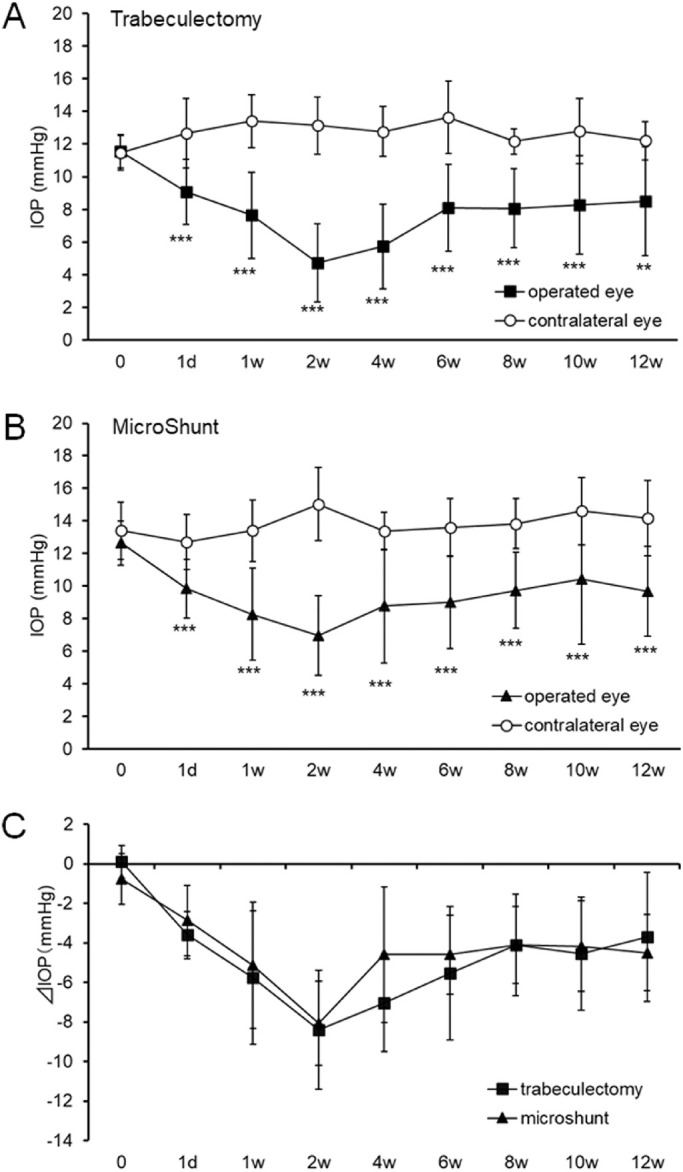
Time course of intraocular pressure (IOP) levels after filtration surgery. (A, B) Time courses of IOP values after trabeculectomy (A) and MicroShunt insertion (B) are shown. Postoperative IOP fluctuations in the same rabbit at up to 12 weeks were recorded. (C) Time course of difference in IOP from the contralateral eye (ΔIOP) is shown. Data are shown as mean ± standard deviation (SD) (*n* = 10). ***P < 0.001, compared to the contralateral eye using Student's *t*-test.

The preoperative IOP in the trabeculectomy and MicroShunt-insertion groups were 11.57 ± 1.02 and 12.63 ± 1.34 mmHg (Figs. 1A and 1B, *n* = 10 per group), respectively. In the trabeculectomy group, the postoperative IOPs were 9.07 ± 2.00, 7.63 ± 2.63, 4.73 ± 2.40, 5.73 ± 2.59, 8.10 ± 2.66, 8.07 ± 2.40, 8.27 ± 3.00, and 8.50 ± 3.33 mmHg at one day and 1, 2, 4, 6, 8, 10, and 12 weeks, respectively (Fig. 1A); in the MicroShunt group, the corresponding IOP values were 9.83 ± 1.81, 8.27 ± 2.82, 6.97 ± 2.44, 8.77 ± 3.50, 9.00 ± 2.84, 9.73 ± 2.35, 10.43 ± 4.01, and 9.67 ± 2.75 mmHg, respectively (Fig. 1B). In both groups, there were significant differences at all postoperative time points compared to the IOP of the contralateral eye (Figs. 1A and 1B). Comparing the IOP of the operated eye between the two groups, the preoperative IOP in the trabeculectomy group was slightly lower than that in the MicroShunt group, but there was no statistical difference (*P* = 0.0601). The postoperative IOP at four weeks in the trabeculectomy group was significantly lower than that in the MicroShunt group (*P* = 0.0407). There were no significant differences in the postoperative IOP between the two groups at other time points. Next, the difference in IOP between the operative eye and contralateral eye (ΔIOP, right eye – left eye) was evaluated. The preoperative ΔIOPs of the trabeculectomy and MicroShunt-insertion groups were 0.10 ± 0.82 and −0.77 ± 1.30 mmHg, respectively (Fig. 1C). In the trabeculectomy group, the postoperative ΔIOP values at each time point (one day and 1, 2, 4, 6, 8, 10, and 12 weeks) were −3.60 ± 1.19, −5.77 ± 3.37, −8.40 ± 3.02, −7.03 ± 2.47, −5.53 ± 3.37, −4.1 ± 2.57, −4.53 ± 2.86, and −3.70 ± 3.28 mmHg. In the MicroShunt group, the corresponding values were −2.87 ± 1.79, −5.13 ± 3.20, −8.07 ± 2.14, −4.60± 3.42, −4.60 ± 1.99, −4.10± 1.94, −4.17 ± 2.28, and −4.50 ± 1.93 mmHg. There were no significant differences in the ΔIOP between the two groups (Fig. 1C) at any time point.

### Bleb Appearance

**Figure 2.**
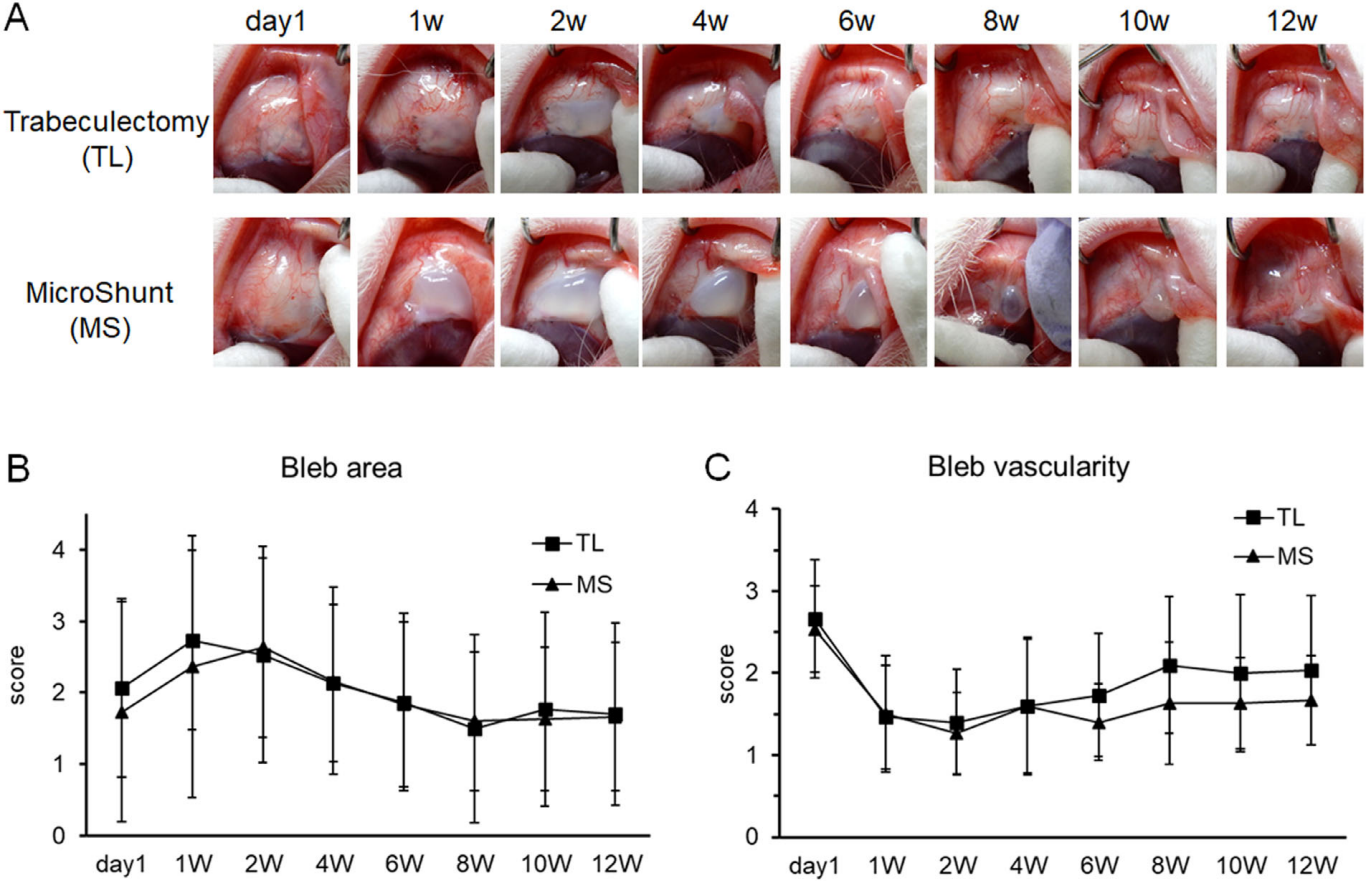
Time course of bleb appearance. (A) The photographs show the change in bleb appearance in the same rabbit over time. The *upper row* is the trabeculectomy-operated eye and the *lower row* is the MicroShunt-inserted eye. (B, C) The area and vascularity of blebs were scored using our own evaluation criteria (Supplementary Figures S1 and S2) based on the Moorfields Bleb Grading System. Data are shown as mean ± SD (*n* = 10). TL, trabeculectomy; MS, MicroShunt insertion.

Representative bleb appearances of each group are shown in Figure 2A. Similar avascular cystic blebs were observed in both groups. The changes in bleb area were similar to the changes in the IOPs of the two groups (Fig. 2B). The peak bleb area occurred at one to two weeks after surgery. It then decreased gradually until approximately eight weeks and remained stable until termination of the rabbits at 12 weeks postoperatively. There were no significant differences in bleb area or vascularity between the two groups (Figs. 2B and 2C) at any time point.

### Distribution of α-SMA-Positive Cells in the Filtering Bleb

**Figure 3.**
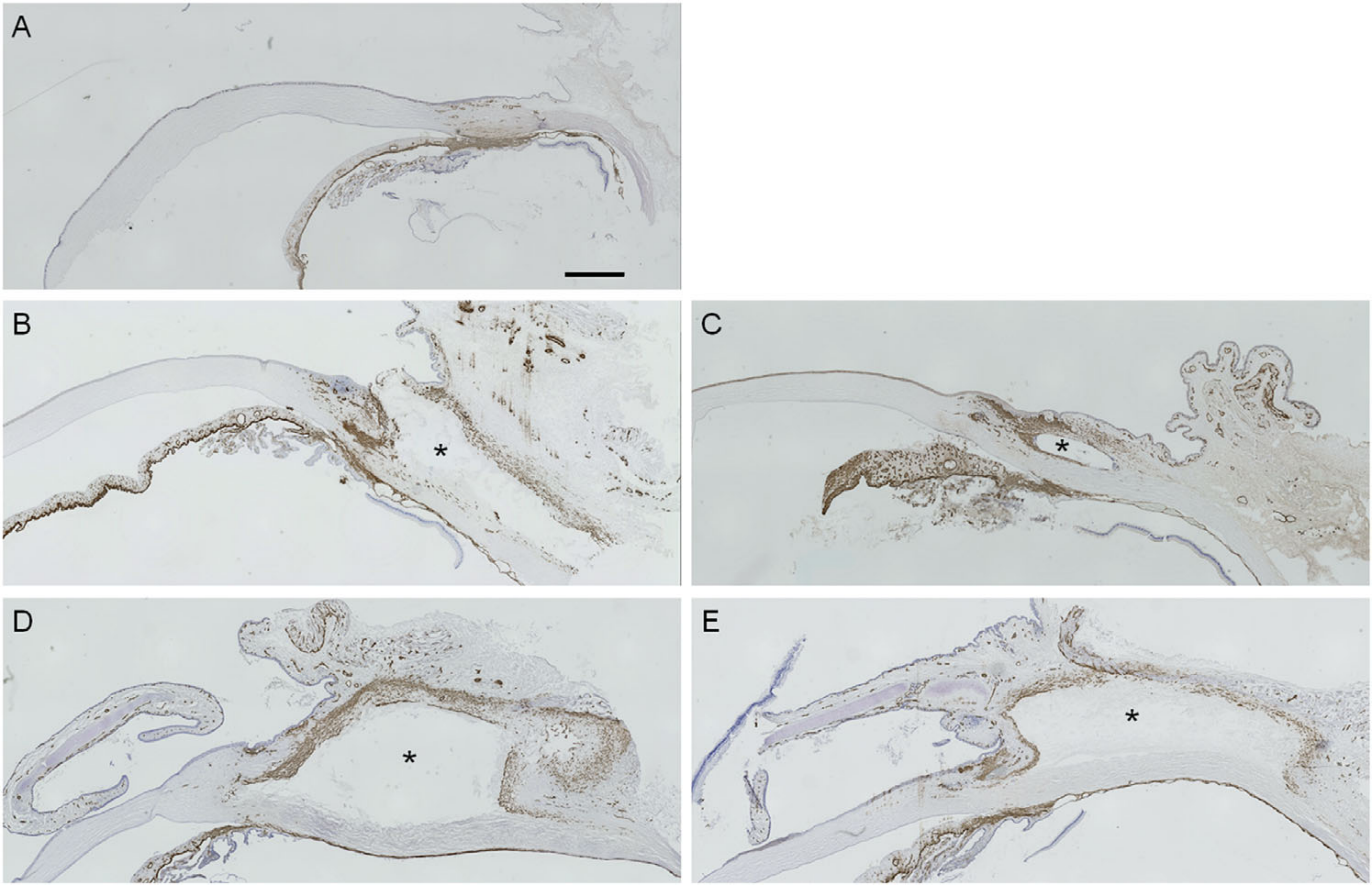
Immunohistochemical analyses of alpha-smooth muscle actin (α-SMA). Normal (A) and operated eyes (trabeculectomy; B, D, MicroShunt insertion; C, E) were stained using α-SMA antibodies at 2 weeks (B, C) and 12 weeks (D, E) after surgery. Immunostaining was performed on three or four eyes at all time points. *Location of the filtering bleb. A 1-mm scale bar is displayed in A. All figures are shown at the same magnification.

As α-SMA-positive cells are biomarkers of tissue scarring, we evaluated the distribution of α-SMA positive cells at four time points (2, 4, 8, and 12 weeks after surgery). In the control eye, α-SMA-positive cells were observed in the ciliary body, iris, and blood vessels of the corneal limbus, but not in the conjunctiva (Fig. 3A). α-SMA-positive cells in the filtering bleb tissue were distributed throughout the bleb inner wall and under the sutures at 2–12 weeks postoperatively for both the trabeculectomy and MicroShunt-insertion groups (Figs. 3B–3E). There were no differences in the distribution of α-SMA-positive cells in the filtering bleb between trabeculectomy and MicroShunt-insertion groups (Fig. 3) at any time point.

### Evaluation of Collagen Type 1 Expression

**Figure 4.**
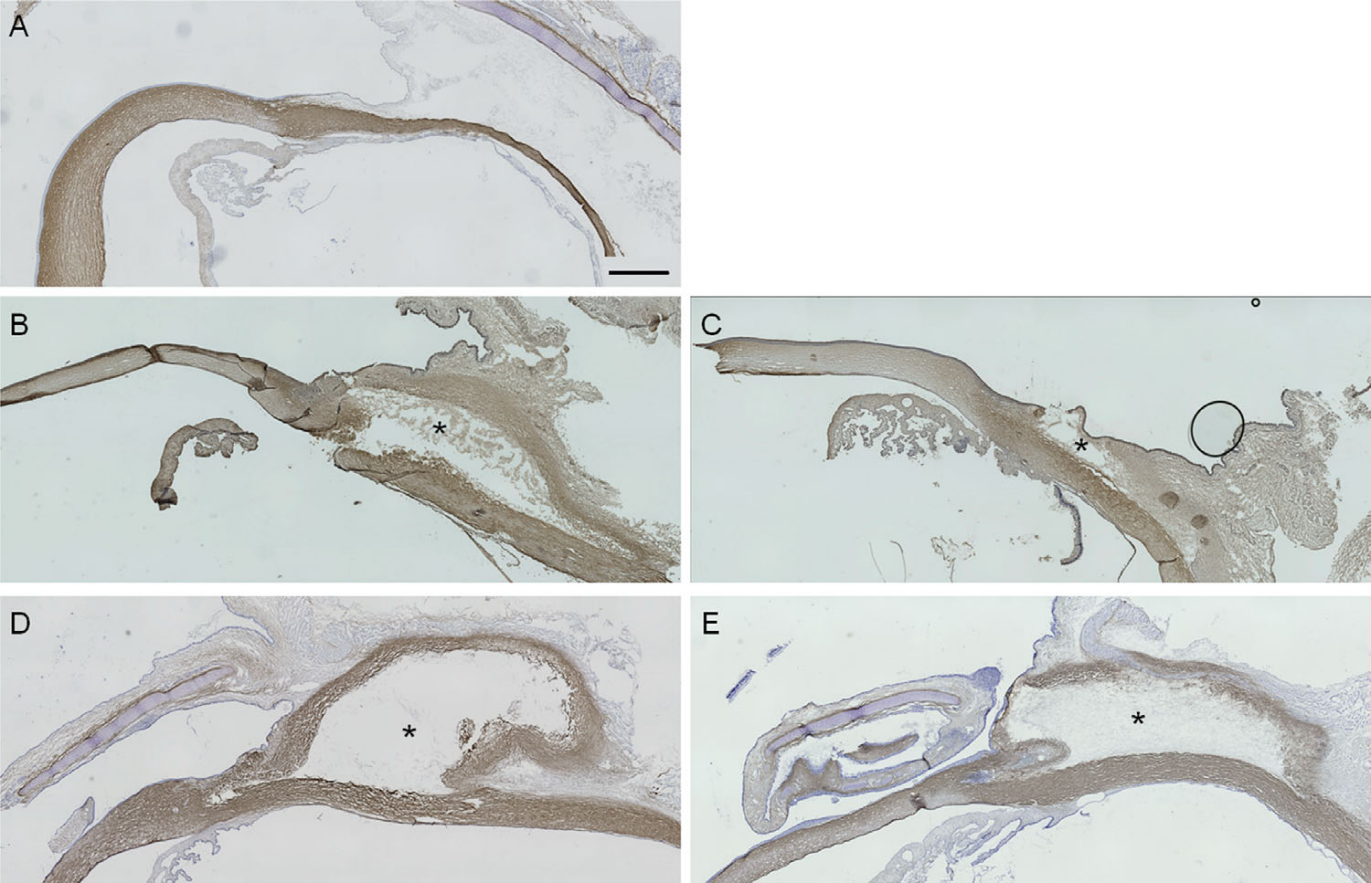
Immunohistochemical analyses of collagen type 1. Normal (A) and operated eyes (trabeculectomy; B, D, MicroShunt insertion; C, E) were stained using collagen type 1 antibody at 2 weeks (B, C) and 12 weeks (D, E) after surgery. Immunostaining was performed on three or four eyes at all time points. *Location of the filtering bleb. A 1-mm scale bar is displayed in A. All figures are shown at the same magnification.

Expression of collagen type 1 in the normal eye was observed in the cornea and sclera, but not in the conjunctiva (Fig. 4A). By contrast, collagen expression in the filtering bleb was apparent in the bleb inner wall at two weeks postoperatively for both the trabeculectomy and MicroShunt-insertion groups (Figs. 4B and 4C), which continued to 12 weeks (Figs. 4D and 4E). There were no differences in collagen expression in the bleb between the trabeculectomy and MicroShunt-insertion groups.

### Aqueous Cytokine Level after Surgery

**Figure 5.**
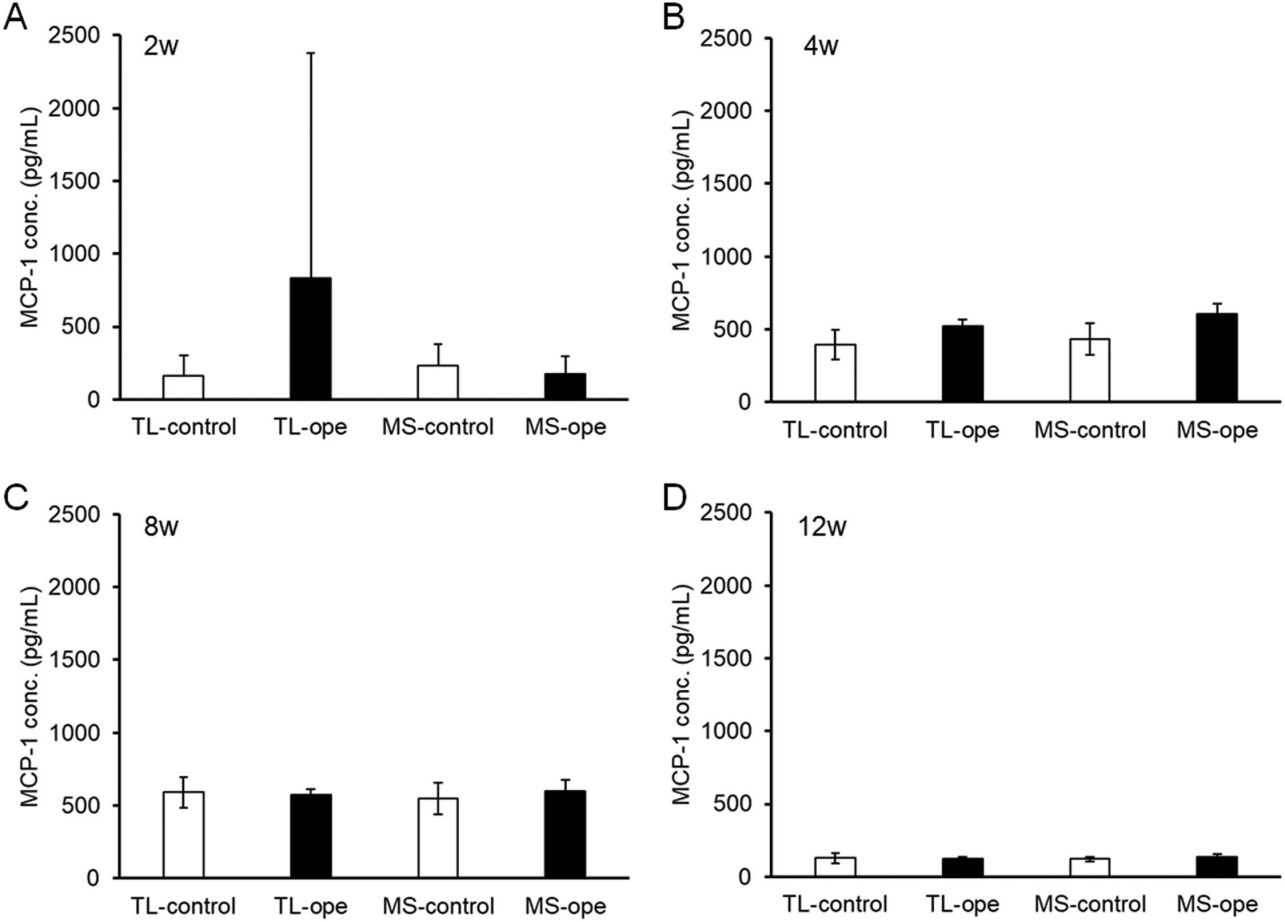
Monocyte chemotactic protein-1 (MCP-1) concentrations in the aqueous humor after filtration surgery. Aqueous humor samples were collected from operated and contralateral eyes at 2 (A), 4 (B), 8 (C), and 12 (D) weeks after surgery. The MCP-1 concentration in the aqueous humor was measured using an enzyme immunosorbent assay (ELISA). Data are shown as mean ± SD (*n* = 4). TL, trabeculectomy; MS, MicroShunt insertion.

**Figure 6.**
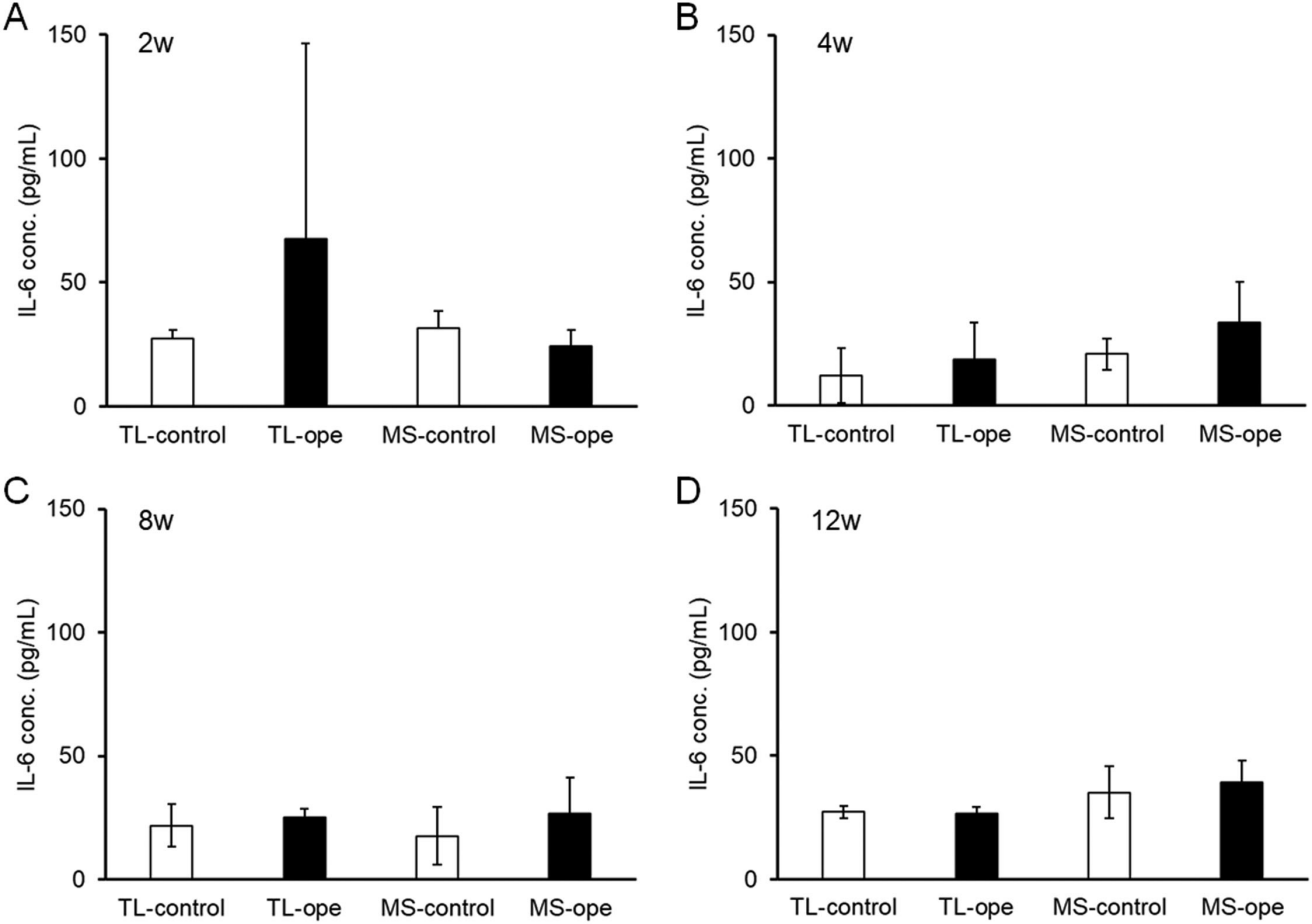
Interleukin-6 (IL-6) concentrations in the aqueous humor after filtration surgery. Aqueous humor samples were collected from operated and contralateral eyes at 2 (A), 4 (B), 8 (C), and 12 (D) weeks after surgery. The IL-6 concentration of the aqueous humor was measured via ELISA. Data are shown as mean ± SD (*n* = 4). TL, trabeculectomy; MS, MicroShunt insertion.

The concentrations of MCP-1 and IL-6 in the aqueous humor after surgery were evaluated using ELISA at 2, 4, 8, and 12 weeks after surgery. It has previously been reported that aqueous concentrations of MCP-1 were elevated at 3, 6, and 12 hours and at two weeks after trabeculectomy.^26^ In this study, MCP-1 concentrations tended to be increased at two weeks after trabeculectomy (831.1 ± 1545 pg/mL), but were not significantly different compared to the contralateral eye (164.4 ± 139.0 pg/mL, *P* = 0.3492; Fig. 5A). At other time points, the concentrations of MCP-1 in the surgical and contralateral eyes were the same (Figs. 5B–5D). On the other hand, no change in MCP-1 concentration was observed after MicroShunt insertion at any time point (Fig. 5). Regarding changes in the concentration of IL-6, the same tendency as that of MCP-1 was observed (Fig. 6). Increased IL-6 concentrations were observed only at two weeks after trabeculectomy (control, 27.2 ± 3.8 pg/mL; operated eye, 67.6 ± 79.0 pg/mL; Fig. 6A), but the increase never reached statistically significant levels (*P* = 0.3781).

### RNA Sequence of Bleb Conjunctiva

**Figure 7.**
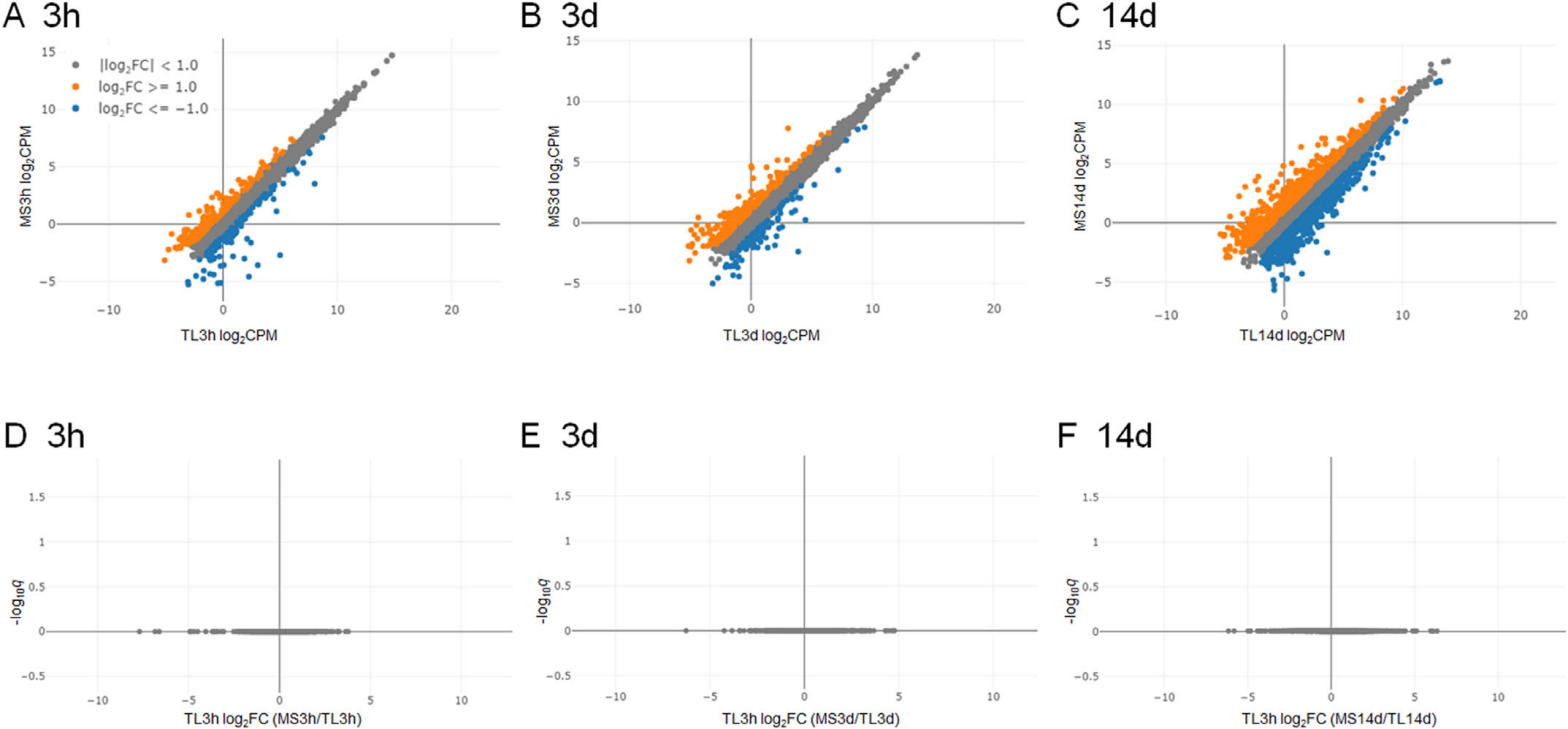
Comparison of MS insertion and TL RNA-seq results by scatter plot (A–C) and volcano plot (D–F). The expression levels of each gene in the bleb conjunctiva at 3 hours (A), 3 days (B), and 14 days (C) after MS and TL are shown using a scatter plot. The vertical and horizontal axes represent the MS and TL expression levels, respectively. *Orange* and *blue dots* indicate genes that were more than twice as high or more than twice as low in MS as TL, respectively. Data are shown as the average value of three eyes. (D, E, and F) Statistical analysis results of changes in the expression levels of each gene between MS and TL at 3 hours (D), 3 days (E), and 14 days (F) after the operation using a volcano plot. No genes differed significantly at any time point.

We evaluated mRNA expression in bleb conjunctiva at 3 hours, 3 days, and 14 days after surgery using RNA sequencing analyses. Of the 14,839 genes for which expression was compared, statistically significant changes after MicroShunt insertion compared to the control were observed in 10,328 (Supplementary Figure S3). The representative results of increased and decreased gene expressions are shown in Supplementary Tables S1–S6. Representative significantly upregulated and downregulated biological processes based on GO analyses using the significantly changed genes after MicroShunt insertion are listed in Supplementary Tables S7–S12. In GO analyses, the expressions of genes involved in the inflammation and immune responses increased at 3 hours, 3 days, and 14 days after surgery. Changes in postoperative gene expression were compared between the MicroShunt and trabeculectomy groups at each time point. Scatter plot analyses showed that the number of genes whose expression levels differed over time tended to increase (Figs. 7A–7C). However, as shown by the volcano plot, no gene showed a statistically significant change in expression level in either of the two groups (Figs. 7D–7F).

## Discussion

The IOP-lowering effects of MicroShunt insertion were confirmed in this 12-week study using a rabbit surgical model. The IOP-lowering effect of the MicroShunt was similar to that of trabeculectomy. This is the first study to demonstrate in a rabbit model that the IOP-lowering effect of the MicroShunt is comparable to that of trabeculectomy. The clinical results of MicroShunt insertion have been reported, and a stable decrease in IOP has been demonstrated for up to one–five years after surgery.^23^^–^^25^^,^^29^ These results imply that MicroShunt insertion may provide long-term IOP reduction.

Trabeculectomy is effective for lowering IOP, but a high level of skill is required for safe performance. MicroShunt insertion, in contrast to trabeculectomy, does not require a scleral incision, sclerectomy, iridectomy, or delicate control of suture tension. Therefore, the MicroShunt procedure is easier and was performed within 15 minutes in this study, compared with 30 minutes for a trabeculectomy. A reduced operation time has the advantage of reducing the burden on both the patient and the surgeon. Moreover, we compared the postoperative inflammatory status of trabeculectomy and MicroShunt insertion based on cytokine concentrations in the aqueous humor. Although the concentrations of IL-6 and MCP-1 did not differ significantly, they tended to be higher at two weeks after trabeculectomy but not after MicroShunt insertion. Although no statistically significant difference was observed, this result suggests that the trabeculectomy procedure could cause inflammation.^26^ No changes in the inflammatory cytokines MCP-1 and IL-6 were observed after MicroShunt insertion, implying that MicroShunt insertion does not elicit an excessive inflammatory reaction in the eye. However, because the number of experiments was small in this study, clinical validation is required.

MicroShunt is constructed from SIBS,^18^ which has been in clinical use as a coating for coronary stents (TAXUS Stent^19^) since 2000 and has been found to be inert and safe.^20^ In this study, the results of bleb appearance, bleb immunohistochemistry, and RNA sequence analyses showed no differences between the MicroShunt and trabeculectomy groups over the 12-week observation period. In our limited number of cases, no reactions (such as excessive inflammation due to MicroShunt implantation) were observed; thus, the device was considered safe. However, given the short observation period of three months and the small number of animals used, further studies are needed to establish long-term safety.

In conclusion, postoperative IOP, bleb appearance, and immunohistochemical analysis results were similar in the trabeculectomy and MicroShunt groups. These results imply that MicroShunt insertion is as effective as trabeculectomy in lowering IOP.

## Supplementary Material

Supplement 1
