# Supplementary material for: Intraocular Pressure-Lowering Effects of Trabeculectomy Versus MicroShunt Insertion in Rabbit Eyes
Source: Transl Vis Sci Technol. 2021 Aug 6;10(9):9. doi: 10.1167/tvst.10.9.9 (PMC8354029; doi:10.1167/tvst.10.9.9)
Supplement: Supplement 1 [file tvst-10-9-9_s001.pdf]

Bleb area score

0: no bleb
1: very small
2: small
3: medium
4: large

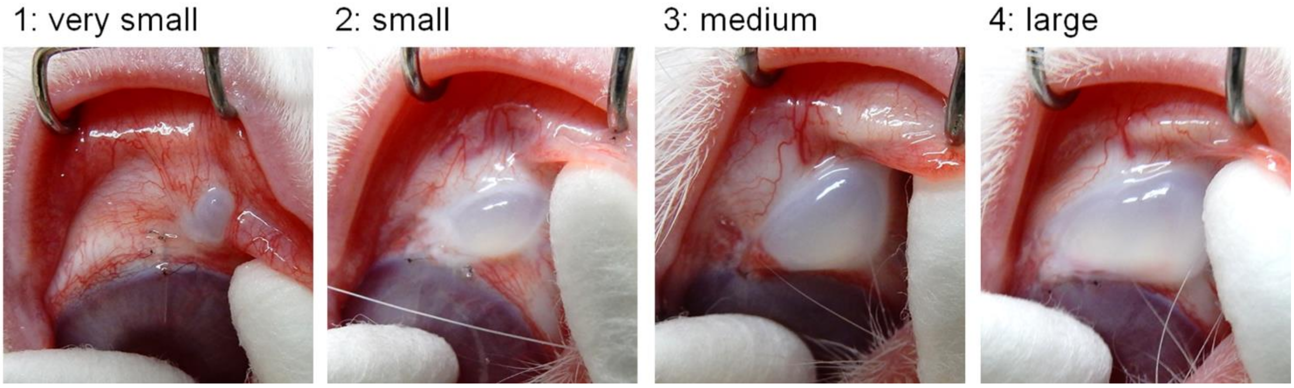

Supplementary Figure S1. Bleb area score criteria.

Bleb vascularity score

1: avascular
2: normal vascularization
3: mild vessel inflammation
4: moderate or severe inflammation

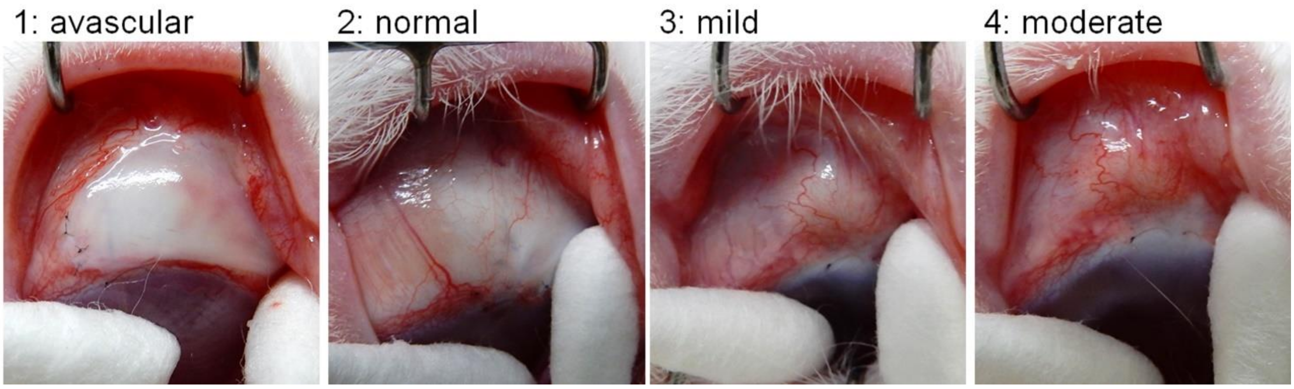

Supplementary Figure S2. Bleb vascularity score criteria.

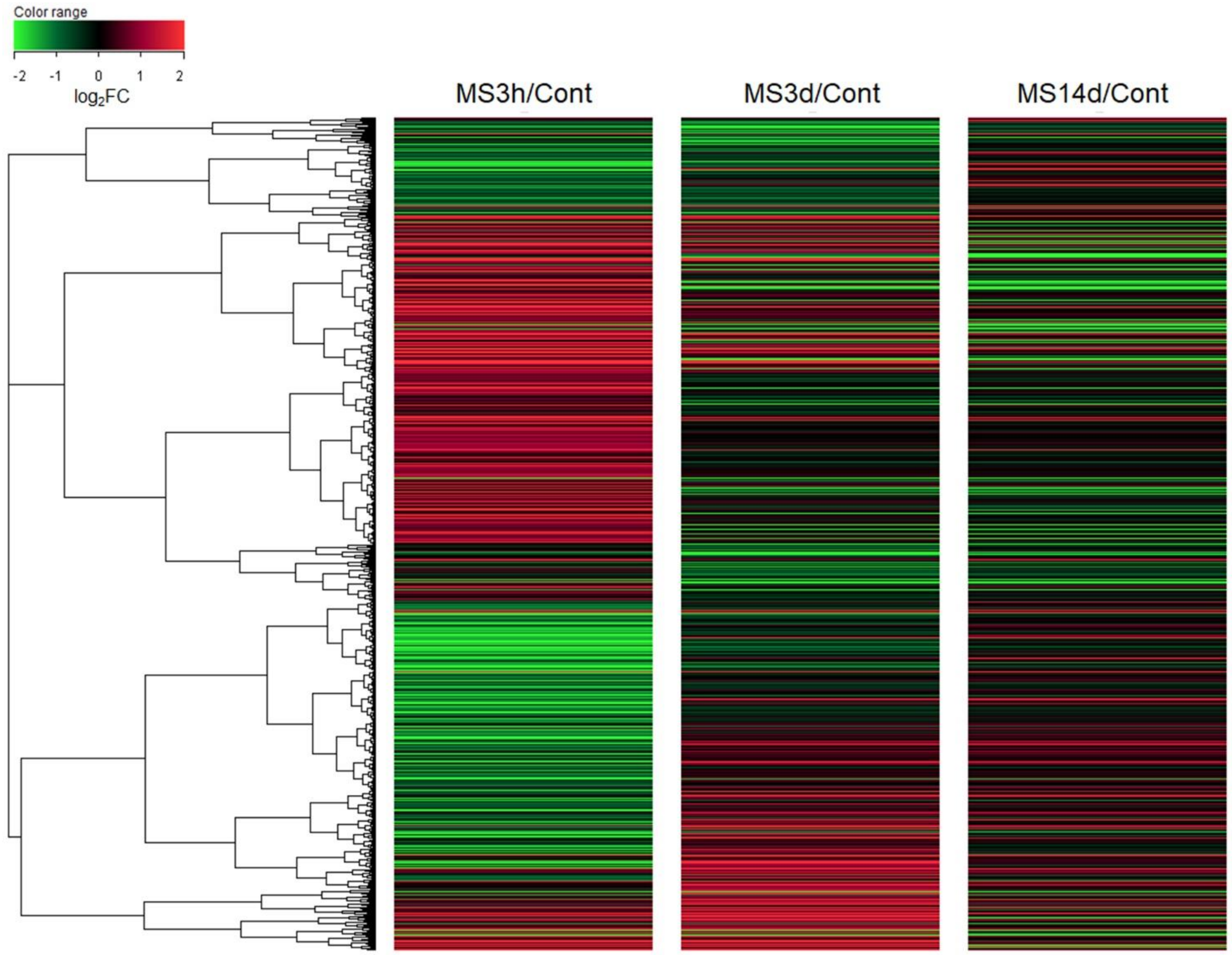

Supplementary Figure S3. Hierarchy-clustered heatmap image of genes that changed significantly in bleb conjunctiva after MicroShunt insertion (MS in figure labeling). Of the 14,839 genes with comparable expression, statistically significant changes compared to the control occurred in 10,328 after MS insertion. Data are shown as log2 FC (FC; fold change, n = 3).

Supplementary Table S1: Top 30 genes with increased expression at 3-hour (3h) after MicroShunt (MS) insertion and fold-changes of these genes at 3h after trabeculectomy (TL)

Gene Symbol	Fold change [MS3h/cont]	Fold change [TL3h/cont]
IL6	6274.3	6615.9
IL20	3601.4	4976.7
DHRS9	1847.7	2111.6
CXCL8	925.5	1334.5
SERPINE1	887.2	1220.5
FOSL1	809.2	953.8
IL23A	744.8	715.7
FGF23	699.7	977.1
PPBP	683.6	908.8
IL36G	615.0	716.2
KRT16	596.6	835.6
ARG1	568.6	817.2
MMP10	567.8	612.1
FPR2	509.1	772.0
MMP3	466.3	338.9
CCL2	452.1	708.6
NTS	412.1	311.4
HRH4	397.1	176.0
PTX3	319.3	589.5
GPR3	312.0	239.2
CCR1	310.0	382.5
LOC108175374	268.6	92.0
IL1A	241.5	429.6
THBS1	235.0	304.0
ABCB11	227.1	414.0
C13H1orf162	226.7	365.9
GNG4	216.6	271.4
IL1B	215.1	415.3
TNFAIP6	198.6	255.2
LOC100347441	186.2	129.4

Supplementary Table S2: Top 30 genes with increased expression at 3-day (3d) after MicroShunt (MS) insertion and fold-changes of these genes at 3d after trabeculectomy (TL)

Gene Symbol	Fold change [MS3d/cont]	Fold change [TL3d/cont]
MMP13	7997.4	9940.6
MMP3	2253.4	1891.7
LOC103351517	1364.0	1024.4
PPBP	936.9	1522.7
PLA2G2F	788.6	661.3
FGF23	783.6	875.0
IL1A	359.4	429.3
OTOG	335.3	257.2
LOC103346278	299.1	282.0
LOC100358922	295.4	237.6
LOC100357363	294.2	648.1
IL20	278.5	413.2
CDSN	267.4	-
MMP9	260.4	436.9
KRT16	247.1	270.6
DPYSL4	244.4	135.0
LOC100347441	223.4	357.7
RSPO2	210.0	249.3
SERPINB13	209.4	179.3
LOC100351904	186.0	192.7
DCDC2C	179.7	-
TNC	168.1	360.0
PRG4	166.5	184.6
SCGB3A2	164.1	36.9
COCH	157.0	127.2
THEM5	154.1	49.1
MUC17	151.2	114.4
MMP25	146.7	90.5
FGFBP2	135.9	58.2
BPI	134.2	77.4

Supplementary Table S3: Top 30 genes with increased expression at 14-day (14d) after MicroShunt (MS) insertion and fold-changes of these genes at 14d after trabeculectomy (TL)

Gene Symbol	Fold change [MS14d/cont]	Fold change [TL-14d/cont]
TNC	1041.2	71.9
MMP13	717.9	158.5
R3HDML	524.9	-
LOC100358018	521.6	-
RSPO2	485.0	71.8
ANKRD1	457.1	135.2
MMP3	454.3	31.8
IBSP	421.1	220.0
CNGA3	387.9	95.3
MMP12	301.7	193.5
LOC100357363	243.0	33.4
LOC103347109	184.8	-
LOC103351517	176.8	25.5
LOC108175368	176.7	19.8
MMP9	172.9	92.0
OTOG	162.7	18.1
PPBP	152.5	102.5
LRRC15	125.8	40.9
LOC100349650	123.3	-
LOC103346278	117.8	9.4
EDIL3	110.6	15.0
UPK3A	108.9	41.2
DCDC2C	103.4	-
PGER2	102.8	12.7
WNT3	96.8	8.7
THBS1	96.0	25.3
LOC100344039	93.6	-
ZBED2	84.7	-
LOC100354881	84.5	13.0
ACAN	83.2	64.4

Supplementary Table S4: Top 30 genes with decreased expression at 3-hour (3h) after MicroShunt (MS) insertion and fold-changes of these genes at 3h after trabeculectomy (TL)

Gene Symbol	Fold change [MS3h/cont]	Fold change [TL3h/cont]
SMTNL2	0.0116	-
KLHL14	0.0213	0.0132
C13H1orf194	0.0219	0.0581
FOXN1	0.0222	0.0758
ADARB2	0.0259	0.0818
LOC103351661	0.0264	-
SMC1B	0.0291	0.1175
EDAR	0.0308	0.1055
PCDH8	0.0310	0.0414
TMEM27	0.0332	-
LNP1	0.0373	-
FAT2	0.0375	0.0563
LOC100343059	0.0416	0.0407
LOC100344261	0.0436	-
LOC100346910	0.0443	0.0897
CLDN8	0.0448	0.0942
LOC103347128	0.0467	0.0524
FBXO43	0.0482	0.1512
LOC100349939	0.0514	0.0116
LOC100348313	0.0524	0.1424
LOC100357811	0.0530	0.0357
RDH12	0.0547	0.1147
FAM167A	0.0550	0.0464
RASGRF1	0.0557	0.2815
LOC103352092	0.0581	0.0594
PRR11	0.0592	0.0298
CENPA	0.0608	0.0587
PPARGC1A	0.0612	0.0831
TRIM46	0.0613	0.0497
SLC16A12	0.0629	0.0645

Supplementary Table S5: Top 30 genes with decreased expression at 3-day (3d) after MicroShunt (MS) insertion and fold-changes of these genes at 3d after trabeculectomy (TL)

Gene Symbol	Fold change [MS3d/cont]	Fold change [TL3d/cont]
LOC100353821	0.0014	0.0003
CYP2A11	0.0033	0.0068
NKAIN3	0.0069	0.0046
OPCML	0.0072	0.0156
LOC100349939	0.0082	0.0058
ERBB4	0.0101	0.0149
LOC100342572	0.0106	0.0101
MPZ	0.0114	-
MYOC	0.0120	0.0069
NEB	0.0129	-
CCKBR	0.0130	0.0381
C13H1orf194	0.0132	0.0221
SEC14L3	0.0146	-
GRM7	0.0149	-
LOC103350283	0.0173	-
LOC100356756	0.0197	-
SMC1B	0.0207	0.1123
S100B	0.0214	-
KCNJ3	0.0215	-
NRG3	0.0234	-
GRIK1	0.0236	-
LOC100351425	0.0239	0.0200
CYP4A7	0.0241	0.0389
PTN	0.0243	0.0122
LOC108178096	0.0246	0.1026
PLCXD3	0.0257	-
MAK	0.0268	0.0975
DPP6	0.0280	0.0293
PCDH8	0.0285	0.0221
ZP4	0.0306	-

Supplementary Table S6: Top 30 genes with decreased expression at 14-day (14d) after MicroShunt (MS) insertion and fold-changes of these genes at 14d after trabeculectomy (TL)

Gene Symbol	Fold change [MS14d/cont]	Fold change [TL-14d/cont]
LOC100353821	0.0039	0.0385
LOC100351425	0.0045	0.0330
GRIK1	0.0048	-
MPZ	0.0052	-
CD5L	0.0054	-
SYT13	0.0059	-
MYOC	0.0067	0.2054
CNTN3	0.0073	-
CDH18	0.0082	0.3690
PTN	0.0086	0.6214
PEG3	0.0086	-
ZP4	0.0090	-
COL9A3	0.0097	-
SCN7A	0.0101	-
LOC100342572	0.0116	0.0381
SOD3	0.0118	-
LOC103346973	0.0132	-
OPCML	0.0144	0.0230
LOC100359112	0.0147	-
SLC24A2	0.0147	0.1997
GFAP	0.0151	-
FRZB	0.0169	-
LOC108178580	0.0173	-
APOD	0.0174	-
TCEAL6	0.0179	-
MAK	0.0180	1.0035
SYPL2	0.0187	-
SERPINA12	0.0191	-
LOC100354078	0.0198	0.1089
DLK1	0.0200	0.1206

Supplementary Table S7: Top 10 GO biological processes of increased genes at 3-hour after MicroShunt insertion

GO ID	GO Term	Count	PValue
GO:0006954	inflammatory response	62	<0.00001
GO:0006955	immune response	60	<0.00001
GO:0008284	positive regulation of cell proliferation	44	0.00494
GO:0001525	angiogenesis	35	<0.00001
GO:0010628	positive regulation of gene expression	32	0.00372
GO:0045766	positive regulation of angiogenesis	29	<0.00001
GO:0030335	positive regulation of cell migration	25	0.00221
GO:0070374	positive regulation of ERK1 and ERK2 cascade	25	0.02318
GO:0008360	regulation of cell shape	24	0.00017
GO:0001666	response to hypoxia	23	0.00005

Supplementary Table S8: Top 10 GO biological processes of decreased genes at 3h after MicroShunt insertion

GO ID	GO Term	Count	PValue
GO:0007165	signal transduction	34	0.02767
GO:0042384	cilium assembly	27	<0.00001
GO:0046777	protein autophosphorylation	23	0.04074
GO:0006260	DNA replication	21	0.00001
GO:0035023	regulation of Rho protein signal transduction	20	0.00064
GO:0007018	microtubule-based movement	19	0.00002
GO:0000724	double-strand break repair via homologous recombination	17	0.00033
GO:0001843	neural tube closure	17	0.00072
GO:0007059	chromosome segregation	16	<0.00001
GO:0007224	smoothened signaling pathway	16	0.00173

Supplementary Table S9: Top 10 GO biological processes of increased genes at 3d after MicroShunt insertion

GO ID	GO Term	Count	PValue
GO:0006955	immune response	59	<0.00001
GO:0006954	inflammatory response	53	<0.00001
GO:0008284	positive regulation of cell proliferation	33	0.01777
GO:0070374	positive regulation of ERK1 and ERK2 cascade	29	0.00001
GO:0045766	positive regulation of angiogenesis	26	0.00000
GO:0010628	positive regulation of gene expression	25	0.00786
GO:0008360	regulation of cell shape	24	<0.00001
GO:0071222	cellular response to lipopolysaccharide	23	<0.00001
GO:0045087	innate immune response	23	0.00029
GO:0030335	positive regulation of cell migration	22	0.00066

Supplementary Table S10: Top 10 GO biological processes of decreased genes at 3d after MicroShunt insertion

GO ID	GO Term	Count	PValue
GO:0007165	signal transduction	19	0.03032
GO:0007605	sensory perception of sound	15	0.00045
GO:0043065	positive regulation of apoptotic process	14	0.01524
GO:0051260	protein homooligomerization	13	0.03819
GO:0043524	negative regulation of neuron apoptotic process	11	0.02712
GO:0007224	smoothened signaling pathway	10	0.00378
GO:0007156	homophilic cell adhesion via plasma membrane adhesion molecules	10	0.02081
GO:0071230	cellular response to amino acid stimulus	8	0.00901
GO:0051965	positive regulation of synapse assembly	8	0.01973
GO:0007018	microtubule-based movement	8	0.02752

Supplementary Table S11: Top 10 GO biological processes of increased genes at 14d after MicroShunt insertion

GO ID	GO Term	Count	PValue
GO:0006955	immune response	44	<0.00001
GO:0006954	inflammatory response	40	<0.00001
GO:0070374	positive regulation of ERK1 and ERK2 cascade	22	<0.00001
GO:0045087	innate immune response	18	0.00006
GO:0010628	positive regulation of gene expression	18	0.00428
GO:0045766	positive regulation of angiogenesis	16	0.00004
GO:0007155	cell adhesion	16	0.00438
GO:0006935	chemotaxis	15	<0.00001
GO:0030335	positive regulation of cell migration	14	0.00517
GO:0030593	neutrophil chemotaxis	13	<0.00001

Supplementary Table S12: Top 10 GO biological processes of decreased genes at 14d after MicroShunt insertion

GO ID	GO Term	Count	PValue
GO:0007165	signal transduction	22	0.00255
GO:0001525	angiogenesis	17	0.00025
GO:0007605	sensory perception of sound	14	0.00108
GO:0008152	metabolic process	13	0.02194
GO:0051260	protein homooligomerization	13	0.03060
GO:0030198	extracellular matrix organization	12	0.00160
GO:0007156	homophilic cell adhesion via plasma membrane adhesion molecules	10	0.01708
GO:0001666	response to hypoxia	9	0.02925
GO:0002062	chondrocyte differentiation	8	0.00075
GO:0001570	vasculogenesis	8	0.00754
